# β-catenin activation drives thymoma initiation and progression in mice

**DOI:** 10.18632/oncotarget.4368

**Published:** 2015-06-08

**Authors:** Chih-Chia Liang, Tsai-Ling Lu, Yi-Ru Yu, Li-Ru You, Chun-Ming Chen

**Affiliations:** ^1^ Department of Life Sciences and Institute of Genome Sciences, National Yang-Ming University, Taipei, Taiwan; ^2^ Institute of Biochemistry and Molecular Biology, National Yang-Ming University, Taipei, Taiwan; ^3^ VYM Genome Research Center, National Yang-Ming University, Taipei, Taiwan; ^4^ Taiwan Mouse Clinic-National Phenotyping Center, Taipei, Taiwan

**Keywords:** thymic epithelium, thymus, β5t, cytokeratin 5, p63, AIRE

## Abstract

Thymoma is the most commonly identified cancer in the anterior mediastinum. To date, the causal mechanism that drives thymoma progression is not clear. Here, we generated K5-ΔN64Ctnnb1/ER^T2^ transgenic mice, which express an N-terminal deletion mutant of β-catenin fused to a mutated ligand-binding domain of estrogen receptor (ER^T2^) under the control of the bovine cytokeratin 5 (K5) promoter. The transgenic mouse lines named Tg1 and Tg4 were characterized. Forced expression of ΔN64Ctnnb1/ER^T2^ in the Tg1 and Tg4 mice developed small thymoma lesions in response to tamoxifen treatment. In the absence of tamoxifen, the Tg1 mice exhibited leaky activation of β-catenin, which activated the TOP-Gal transgene and Wnt/β-catenin-targeted genes. As the Tg1 mice aged in the absence of tamoxifen, manifest thymomas were found at 10-12 months. Interestingly, we detected loss of AIRE and increase of p63 in the thymomas of Tg1 mice, similar to that observed in human thymomas. Moreover, the β5t protease subunit, which was reported as a differential marker for human type B3 thymoma, was expressed in the Tg1 thymomas. Thus, the Tg1 mice generated in this study accurately mimic the characteristics of human thymomas and may serve as a model for understanding thymoma pathogenesis.

## INTRODUCTION

The thymus is an organ that provides a specialized microenvironment for the thymocyte development. The thymic microenvironment consists of a variety of cell types, including the thymic epithelial cells (TECs). TECs can be further anatomically sub-categorized as cortical TECs (cTECs) and medullary TECs (mTECs). cTECs are essential for the positive selection of developing thymocytes, while mTECs establish self-tolerance of selected thymocytes [1]. cTECs and mTECs can be distinguished by differentially expressed cytokeratin profiles in the adult human thymus [2]. In mice, cytokeratin 8 (K8) and cytokeratin 5 (K5) are also commonly used for histological examination of TEC populations in the cortex and medulla of the postnatal thymus [3-6]. In addition, mTECs express p63 [6, 7], AIRE [6, 8-12] and UEA-1 [5, 13-15], whereas cTECs express β5t [12, 16, 17] and Ly51 [6, 12]; these additional markers can be used to further sub-classify EpCAM^+^ TEC populations in the physiological or pathological thymus.

Tumors arising from TECs are called thymomas or thymic carcinomas and account for 0.2%-1.5% of all cancers types [18, 19]. Classification of thymic epithelial tumors is mainly based on the histological features of the tumor, including epithelial cell morphology, rich or scant thymocytes, cellular atypia, and invasiveness. Due to the rarity of thymic epithelial tumors, the etiological factors that contribute to the development of thymomas remain unclear.

According to the World Health Organization (WHO), the histological classification of thymic epithelial tumors is subdivided into type A thymomas, type B thymomas, type AB mixed, and type C (or thymic carcinoma) [20]. The type A thymoma is comprised of spindle or oval neoplastic cells with few or no lymphocytes. Type B thymoma has predominantly large polygonal or round epithelial cells. Type B thymomas can be further subdivided into types B1, B2, or B3 based on the morphology of neoplastic epithelial cells and the extent of lymphocytic infiltration. The type B1 thymoma is a lymphocyte-rich tumor, and the type B2 thymoma exhibits an increased number of clustered or scattered neoplastic epithelial cells among a large population of infiltrated lymphocytes. The type B3 thymoma is composed of predominantly epithelial cells with few or no lymphocytes. In addition, an unique perivascular space pattern is commonly present in the types B2 and B3 thymomas. Type AB mixed thymoma is similar to type A thymoma with lymphocyte-rich areas. Type C thymoma (or thymic carcinoma) is a rare tumor type that displays higher invasiveness and metastatic potential [18]. The WHO classification system appears to reflect the oncogenic potential of thymomas and correlates with the clinical stages of patients with thymoma [21, 22]; however, the small number of cases has impeded the ability to establish statistical significance between the WHO histological typing system and clinical outcomes [23]. Therefore, further studies on the biological behavior, histopathological features, prognosis, therapeutic strategies, and follow-up information of thymomas are highly warranted.

Previous molecular genetic studies have identified loss of heterozygosity (LOH) at the adenomatous polyposis coli (*APC)* locus was associated with the type B thymomas [24, 25]. The critical role of APC-mediated signaling in thymic epithelial differentiation and carcinogenesis was further explored in mice by conditional ablation of the *Apc* gene using K14-Cre [26]. Interestingly, loss of Apc in K14-expressing TECs caused thymic atrophy and squamous metaplasia, suggesting that *Apc* is required for thymic epithelial differentiation and development [26]. APC is known to play a key role in regulating stability and nuclear entry of β-catenin, which is a crucial mediator of the canonical Wnt signaling pathway involved in cell-fate determination, proliferation, embryonic development, and cancer progression [27]. Forced expression of stabilized β-catenin in embryonic Foxn1-expressing thymic epithelial primordium leads to the loss of thymic epithelial identities and transdifferentiates into epidermal cell-fate [28]. However, the thymoma phenotype was not described in either study [26, 28]. Defects in early thymic organogenesis likely preclude the long-term monitoring of thymoma development.

In our previous study, we demonstrated that loss of β-catenin in a K5-expressing TEC lineage resulted in thymic atrophy [6]. Loss of β-catenin leads to aberrant differentiation of TECs and subsequent thymocyte development defects [6]. Thus, β-catenin levels may be critical for the physiological and pathological states of TECs. To examine the role of β-catenin in thymic homeostasis, we generated an inducible transgenic mouse named *K5-ΔN64Ctnnb1/ER^T2^*, which expresses the C-terminal portion of β-catenin fused to a mutated ligand-binding domain of the estrogen receptor (ER^T2^) to control activation of β-catenin in K5-expressing cells using tamoxifen (Tam). Our results with this transgenic line show that ΔN64Ctnnb1/ER^T2^ can be induced by Tam treatment in both TECs and hair follicles. Very importantly, we observed that nuclear localization of β-catenin in TECs promoted thymoma progression. These results indicate that β-catenin is critical for maintaining thymic epithelial homeostasis and driving thymoma progression.

## RESULTS

### Characterization of transgenic mice expressing ΔN64Ctnnb1/ER^T2^ driven by bovine K5 promoter in the thymus and skin

**Figure 1:**
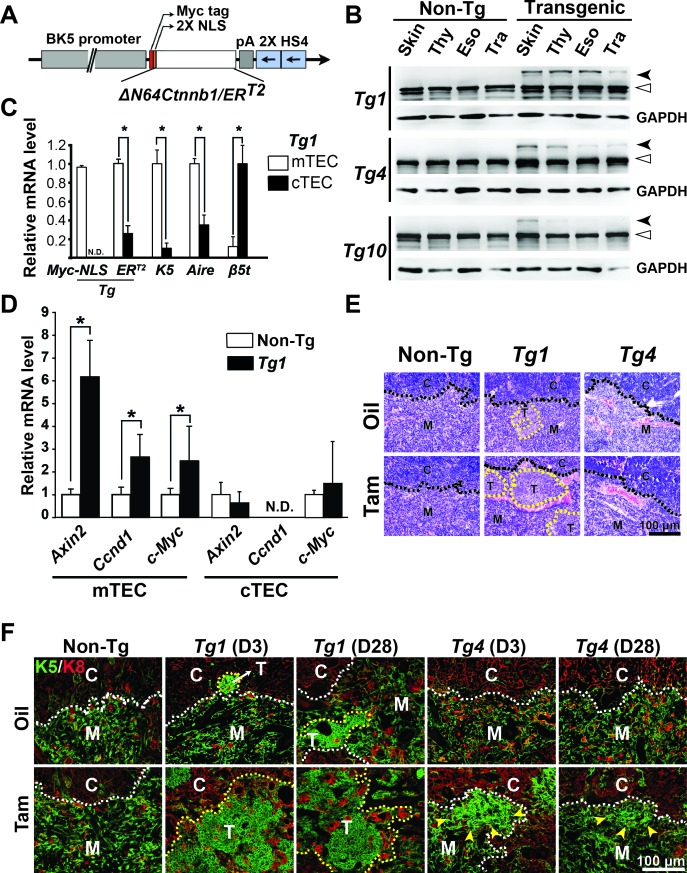
Characterization of Tg(*K5-ΔN64-Ctnnb1/ER*) transgenic mice **A.** Diagram illustrates the construct used for generating the transgenic mice. **B.** Western blot analysis reveals relative expression levels of endogenous β-catenin (open arrowheads) and transgenic ΔN64-Ctnnb1/ER^T2^ fusion protein (close arrowheads) in various organs (skin, thymus, esophagus, and trachea) of Tg1, Tg4, and Tg10 compared to non-transgenic (non-Tg) controls. GAPDH served as an internal control. **C.** Relative expression levels of *ΔN64-Ctnnb1/ER*^T2^ transgene (Myc-NLS and ER^T2^), mTEC-specific *K5* and *Aire*, and cTEC-specific β*5t* expression are determined by RT-qPCR. RNA samples were isolated from the sorted CD45^−^EpCAM^+^UEA1^+^ mTECs and CD45^−^EpCAM^+^Ly51^+^ cTECs of Tg1 thymi. N.D., not detected. **D.** Relative expression levels of Axin2, Ccnd1, and c-Myc are determined by RT-qPCR. RNA was isolated from the sorted mTECs and cTECs of non-Tg or Tg1 thymi. **E.** H&E staining of thymic sections from vehicle (oil)- and Tam-treated non-Tg, Tg1, and Tg4 mice shows notable cortex and medulla areas, referred to as C and M (separated by black dashed lines). Aberrant lesions in the Tg1 thymic sections are depicted by yellow dashed lines at the cortical-medullary junction (CMJ). **F.** Co-immunofluorescent staining of K5 (green) and K8 (red) in the thymic sections from oil- and Tam-treated non-Tg, Tg1, and Tg4 mice for 3 or 28 days shows cTECs at the cortex **C.**, mTECs at medulla (M) (separated by white dashed lines), and aberrant K5-expressing TEC clusters (T; depicted by yellow dashed lines or pointed by arrowheads). Scale bar, 100 μm.

To address the function of stabilized β-catenin in the thymic epithelium of adult mice, *K5-ΔN64Ctnnb1/ER^T2^* mice were generated. These mice harbor an N-terminal, 64-amino-acid truncation of β*-catenin* fused to the tamoxifen (Tam)-inducible ligand-binding domain of estrogen receptor (*ER^T2^*) driven by a bovine K5 promoter (Figure 1A). The expression of ΔN64Ctnnb1/ER^T2^ in various organs of transgenic mouse lines 1, 4, and 10 (hereafter referred to Tg1, Tg4, and Tg10) were detected using an anti-β-Catenin antibody for western blot analysis. We found that Tg1 and Tg4 exhibited comparable amounts of the ΔN64Ctnnb1/ER^T2^ fusion protein in the skin, whereas skin from Tg10 expressed lower levels of ΔN64Ctnnb1/ER^T2^ fusion protein (Figure 1B). In the thymus and the esophagus, we observed higher ΔN64Ctnnb1/ER^T2^ protein levels in the Tg1 mice than in Tg4 or Tg10 mice (Figure 1B). In general, ΔN64Ctnnb1/ER^T2^ expression of Tg10 was lowest among three transgenic lines (Figure 1B). We further examined transgene copy numbers of Tg1 and Tg4 mice using southern blot analysis. We found that Tg1 and Tg4 carried about 8 and 4 copies of the transgene, respectively (Supplementary Figure 1). Thus, Tg1 and Tg4, which had higher transgene expression, were selected for more detailed characterization.

Furthermore, we characterized ΔN64Ctnnb1/ER^T2^ expression and activation in the TECs of the Tg1 mice at 8 weeks of age. After 3 days of Tam administration, thymi were collected and TECs were sorted. Within CD45^−^EpCAM^+^ gated TECs, mTECs and cTECs were analyzed and sorted using cell surface markers UEA1 and Ly51, respectively (Supplementary Figure 2A). We found that the percentages of mTECs (UEA1^+^) and cTECs (Ly51^+^) in CD45^−^EpCAM^+^ gated cells were comparable between non-transgenic control (non-Tg) and Tg1 thymi (Supplementary Figure 2B). Using RT-qPCR, we found that ΔN64Ctnnb1/ER^T2^ expression detected by the transgene-specific primers (Myc-NLS or ER^T2^) was significantly enriched in CD45^−^EpCAM^+^UEA1^+^ mTECs (Figure 1C). As expected, the mTEC-specific genes, K5 and Aire, and cTEC-specific β5t were enriched in the CD45^−^EpCAM^+^UEA1^+^ mTECs and CD45^−^EpCAM^+^Ly51^+^ cTECs, respectively (Figure 1C). In consistent with the transgene expression, we also found that Wnt/β-catenin-targeted genes, such as Axin2, cyclin D1, and c-Myc, were significantly upregulated in the mTECs, but not in the cTECs, of Tam-treated Tg1 mice (Figure 1D). These data suggest that the ΔN64Ctnnb1/ER^T2^ transgene, driven by K5 promoter, is restricted and can be induced by Tam to activate its target genes in mTECs.

Next, we examined several potential phenotypes of TECs using histology and K5/K8 co-immunofluorescence analyses in the presence or absence of Tam administration to 8-week-old Tg1 and Tg4 mice. Microscopically, we found a few small lesions with squamoid appearance and rare lymphocytes in the thymic cortical-medullary junction (CMJ) of vehicle-treated Tg1 mice. These lesions were expanded in the Tam-treated Tg1 thymic CMJ and medulla (Figure 1E, middle). However, we found no overt phenotype in the histological views of thymic medullae from vehicle only (oil-treated) or Tam-treated Tg4 or non-Tg control mice (Figure 1E). We performed K5/K8 co-immunostaining on the thymic sections of non-Tg control, Tg1, and Tg4 mice after vehicle or Tam treatment for 3 or 28 days. In the non-Tg control, we observed K5- and K8-expressing cells predominately in the medulla and cortex, respectively (Figure 1E). In the vehicle-treated Tg1 mice, aberrant K5-expressing cell clusters residing in the CMJ of the thymi were observed (Figure 1F), indicating possible leaky expression of -catenin, leading to sufficient activity to drive thymoma initiation. However, in the vehicle-treated Tg4 mice, the aberrant K5-expressing cell clusters were not detected (Figure 1F). After Tam treatment for 3 days or 28 days in Tg1 and Tg4 mice, expanded K5-expressing squamoid cells were observed, although the aberrantly expanded K5-expressing lesions were smaller in the thymi of Tg4 (Figure 1F). This observation may be due to lower ΔN64Ctnnb1/ER^T2^ transgene expression in the thymus as seen by western blot (Figure 1B).

Next, we examined ΔN64Ctnnb1/ER^T2^ expression in hair cycle re-entry because previous studies have demonstrated that transient β-catenin activation in the skin results in stimulation of resting (telogen) hair follicles to the growing state (anagen phase) [29, 30]. Therefore, we applied Tam topically on the back skin of Tg1 and Tg4 mice and examined hair cycle re-entry. In agreement with these reports [29, 30], we observed that hair follicles had grown into deeper layers of the dermis, with marked expansion of outer root sheet (ORS) cells, in Tg1 and Tg4 mice that received Tam compared to those that received vehicle (EtOH) only (Supplementary Figure 3A). We also examined potential leakage activation of ΔN64Ctnnb1/ER^T2^ fusion protein in the absence of Tam. We observed darker skin, indicating hair follicles in growing state, on the back skin of shaved Tg1 and Tg4 mice compared to non-Tg controls without Tam treatment for 16-20 days (Supplemental Figure 3B), suggesting possible leaky expression and activity of β-catenin. Taken together, our data suggest that ΔN64Ctnnb1/ER^T2^ can be induced by Tam treatment in both TECs and hair follicles in Tg mice, although leaky expression of active ΔN64Ctnnb1/ER^T2^ may be present.

### Monitoring thymomas in leakage activation of β-catenin in K5-expressing TECs in the aged transgenic mice

**Figure 2:**
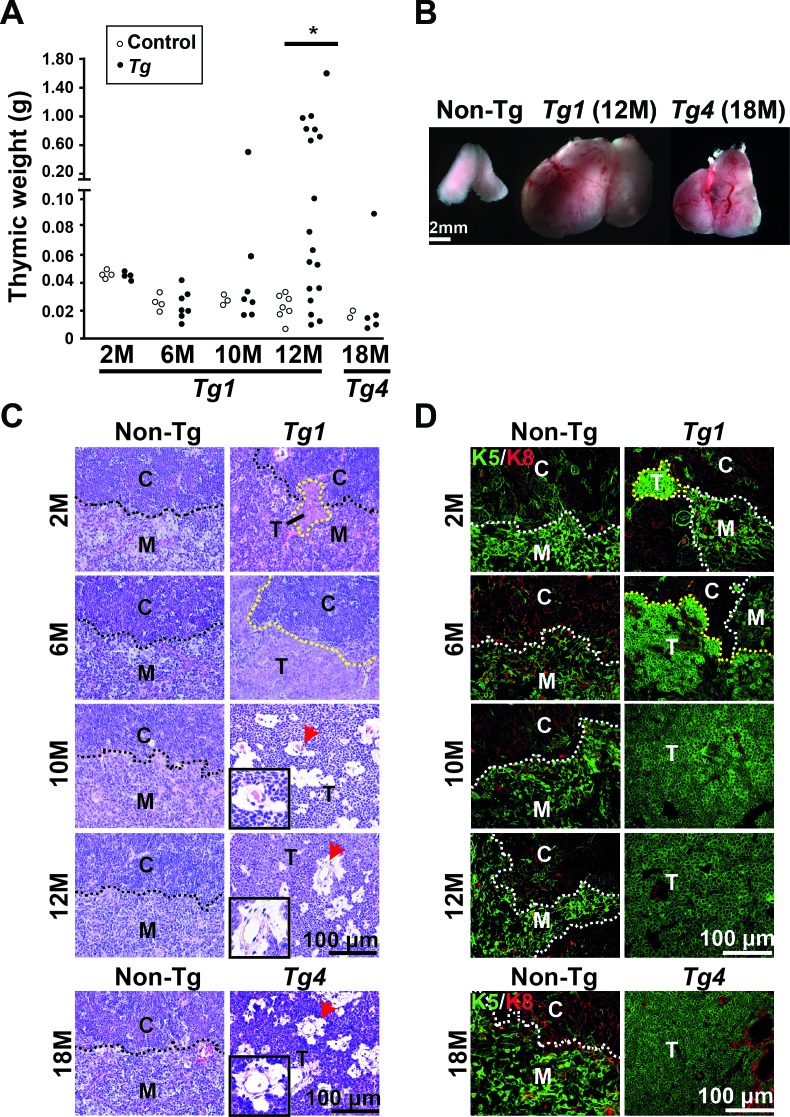
Spontaneous thymoma development in the Tg1 and Tg4 mice in the absence of tamoxifen (Tam) treatment **A.** The thymi of non-Tg and Tg1 mice were weighed at 6, 8, 10, and 12 months of age. The thymi of Tg4 mice and non-Tg littermates were weighed at 18 months of age. Each circle represents an independent mouse. [*], *p* < 0.05; **B.** Representative images of a non-Tg thymus, a spontaneous thymoma developed in one Tg1 mouse at 12 months old, and a spontaneous thymoma developed in one Tg4 mouse at 18 months are shown. **C.** H&E staining of thymic sections reveals non-Tg thymi and thymoma development in Tg1 mice between 6 and 12 months. Lower panels are H&E staining of a non-Tg thymus and a Tg4 thymoma at 18 months. C, cortex; M, medulla; T, tumor lesion; Black dashed lines, CMJ; Red arrows, perivascular space. Insets, high-powered view of perivascular space in thymomas; **D.** Co-immunofluorescent staining reveals the expression of K5 (green) and K8 (red) in the non-Tg thymi and thymoma lesions in the Tg1 (2, 6, 10, and 12 months) and Tg4 thymi (18 months). White dashed lines represent the CMJ. Yellow dashed lines depict the tumor boundary; Bar scale, 100 μm.

Because leaky expression of active ΔN64Ctnnb1/ER^T2^ occurred in our established Tg mice, we monitored spontaneous tumor progression, which might be caused by the activation of β-catenin in the absence of Tam. We found manifest thymic tumors specifically in Tg1 and Tg4 mice but not in the non-Tg littermates (Figure 2A and 2B). The tumor incidence in Tg1 mice increased from 23.6% (2 of 7 mice) at 10 months old to 63.7% (12 of 18 mice) at 12 months old (Figure 2A). Also, one of five Tg4 mice developed manifest thymomas at late onset (18 months) (Figure 2B). The low incidence of thymoma development in Tg4 mice might reflect the lower expression of ΔN64Ctnnb1/ER^T2^ in the Tg4 thymus overall (Figure 1B). These data suggest that a leaky expression of activated β-catenin cause thymoma development in the Tg1 and Tg4 mice without Tam treatment.

We analyzed the thymoma phenotype in Tg1 mice and their non-Tg littermates at 2 months and 6 months of age. Although we could not observed manifest tumors in Tg1 thymi at 2 or 6 months, we observed microscopic thymoma lesions embedded in the CMJ of all Tg1 thymi (*n* = 4) at 2 months or formed an epithelial sheet in the medullary area of all Tg1 thymi (n = 7) at 6 months (2 and 6 months; Figure 2C). At 10 or 12 months, we found that the manifest thymic tumors of Tg1 mice were composed of polygonal epithelial cells with a squamoid appearance and scant lymphocytes (Figure 2C). Notably, perivascular spaces with loose connective tissues appeared between vessels and neoplastic epithelial sheets (10 and 12 months; Figure 2C), resembling the histology characteristics of type B3 thymoma, or atypical thymoma, in humans. Using immunofluorescence staining for K5 and K8, we showed that the early thymoma lesions expressed K5, but not K8 (2 months; Figure 2D). The K5-expressing lesions accumulated and formed a sheet-like pattern at 6 months that seemed to correspond to the pre-lesions of Tg1 thymomas at 10 and 12 months (Figure 2D). These K5-expressing lesions in Tg1 thymi at 6 months of age were comparable to the features of short-term (3 days and 28 days) Tam-induced early lesions in the thymi of Tam-treated Tg1 mice (Figure 2D *versus* Figure 1F). Moreover, Tg4 tumors identified at 18 months exhibited similar histological features and K5 expression as those seen in Tg1 tumors at 10-12 months, indicating that lower transgenic β-catenin activation correlates with slower thymoma progression (Figure 2C and 2D).

In addition, we performed flow cytometry to detect CD45^−^EpCAM^+^ gated UEA1^+^Ly51^−^ mTECs and UEA1^−^Ly51^+^ cTECs in non-Tg thymi and Tg1 thymomas. In the non-Tg thymi, UEA1^+^Ly51^−^ subset was reduced (approximately 2.5-fold) and UEA1^+^Ly51^+^ population was increased (approximately 3.5-fold) in 12-month-old mice compared to those subsets from 6-month-old mice (Supplementary Figure 4A and 4B), suggesting the changes of TEC subsets are associated with age. In the Tg1 thymi at 6 months old, all TEC subsets in CD45^−^EpCAM^+^ cells were comparable to those in non-Tg thymi (Supplementary Figure 4A & 4B). In the thymomas from 12-month-old Tg1 mice, we found that the collagenase could not completely dissociate the tumor fragments, resulting in a collagenase resistant portion (C-res) that could be subsequently dissociated by trypsin. Interestingly, the collagenase-dissociated and C-res subsets in the Tg1 thymomas were composed of higher percentage of UEA1^+^Ly51^−^ subset and lower percentage of UEA1^+^Ly51^+^ population compared to the TEC subsets of thymi from 12-month-old non-Tg mice (Supplementary Figure 4A & 4B). Percentages of UEA1^−^Ly51^+^ subsets were comparable in non-Tg thymi and Tg1 thymoma (Supplementary Figure 4B). Therefore, it is possible that the sustained K5^+^ and UEA1^+^Ly51^−^ subsets in Tg1 thymomas arising from the mTECs, where the ΔN64Ctnnb1/ER^T2^ transgene was expressed, maintained the differentiation characteristics of mTECs during thymoma progression.

### Activated Wnt/β-catenin canonical pathway in the mouse thymomas

**Figure 3:**
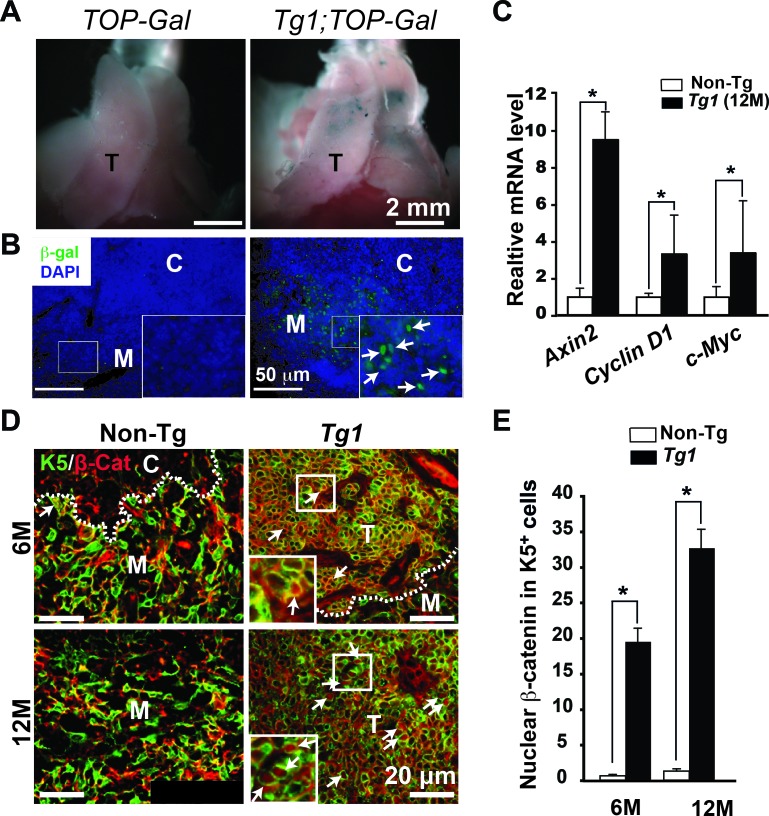
Activated Wnt/β-catenin canonical pathway in the spontaneous Tg1 thymomas **A.** β-galactosidase activity was determined by the whole-mount X-gal staining (blue) in the TOP-Gal and Tg1;TOP-Gal thymi (*n* = 3 each genotype) at 6 months. T, the thymus. **B.** Immunofluoresent staining of anti-β-galactosidase antibody reveals nuclear β-galactosidase expression (green) in Tg1;TOP-Gal, but not in the control (TOP-Gal) thymic medullae. Insets, high-power view of medulla; Arrows indicate nuclear β-galactosidase. DAPI (blue) was used for nuclear counterstaining. **C.** RT-qPCR reveals the relative expression levels of Wnt/β-catenin canonical target genes, such as Axin 2, Cyclin D1 (Ccnd1), and c-Myc, in the CD45^−^EpCAM^+^ sorted TECs from 12-month-old control thymi and Tg1 thymomas (*n* = 3). [*], *p* < 0.05. **D.** Co-immunofluorescent staining reveals the expression of K5 (green) and β-catenin (red) in the thymic sections of control and Tg1 mice at 6 and 12 months. White dashed lines, CMJ; Yellow dashed lines, the microscopic boundary of thymoma; Arrows, nuclear β-catenin; Insets, high-power view of thymoma lesions. **E.** Quantification of nuclear β-catenin in K5-expressing cells in non-Tg thymi and Tg1 thymomas at 6 and 12 months of age (*n* = 3, each group). [*], *p* < 0.05.

To examine whether the Wnt/β-catenin canonical pathway is activated in ΔN64Ctnnb1/ER^T2^ thymomas in the absence of Tam, we introduced the TOP-Gal transgene to allow the measurement of Wnt/β-catenin activation. Using whole-mount X-gal staining, we found that Tg1;TOP-Gal thymi clearly exhibited patched blue staining compared to negatively stained non-Tg littermate (TOP-Gal) thymi at 6 months (Figure 3A). Immunohistochemistry also revealed that the β-galactosidase was specifically detected in Tg1;TOP-Gal thymic medullae, but not in the non-Tg control (Figure 3B). We next examined several targeted genes of Wnt/β-catenin canonical pathway, which should be activated if the ΔN64Ctnnb1/ER^T2^ transgene is functional during thymoma progression. Indeed, Axin2, Cyclin D1 and c-Myc were upregulated in the manifest thymomas compared to the CD45^−^ thymic stromal cells isolated from non-Tg thymi (Figure 3C). Nuclear localization of β-catenin was also observed and significantly increased in the early lesions and manifest thymomas of Tg1 mice at 6 and 12 months of age (Figure 3D and 3E). These data suggest that canonical Wnt/β-catenin signaling is activated in thymomas and drives their progression.

### Compensation of endogenous β-catenin loss by ΔN64Ctnnb1/ER^T2^ transgene for maintaining thymic homeostasis

**Figure 4:**
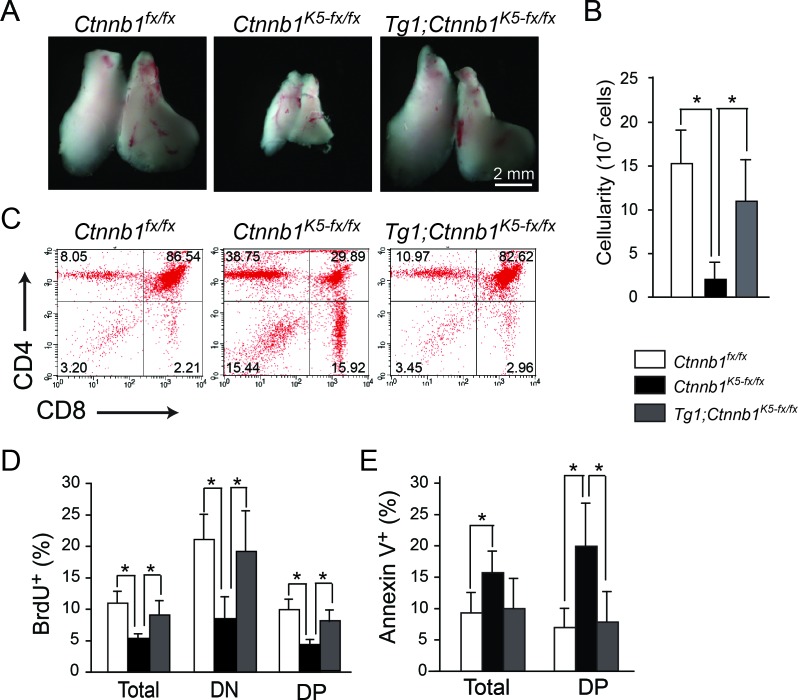
Expression of constitutively activated β-catenin in K5^+^ lineage TECs rescues the thymopoiesis defects observed in Ctnnb1^K5-fx/fx^ thymi **A.** Gross view of the thymi of *Ctnnb1^fx/fx^, Ctnnb1^K5-fx/fx^ and Tg1;Ctnnb1^K5-fx/fx^* mice. The thymi of *Tg1;Ctnnb1^K5-fx/fx^* (right) mice were similar in size to control (*Ctnnb1^fx/fx^*) thymi (left). **B.** Absolute cell numbers in *Ctnnb1^fx/fx^* control (white bar), *Ctnnb1^K5-fx/fx^* (black bar) and *Tg1;Ctnnb1^K5-fx/fx^* thymi (gray bar) on day 10 post-4-OHT injection (*n* = 4 in each genotype). **C.** Representative dot plots show the similar CD4/CD8 expression profiles among developing thymocytes in *Ctnnb1^fx/fx^, Ctnnb1^K5-fx/fx^ and Tg1;Ctnnb1^K5-fx/fx^* thymi on day 10 post-4-OHT injection. **D.** Bar graphs represent the percentages of BrdU-labeled cells among total thymocytes, DN cells, and DP cells. **E.** Bar graphs represent the percentages of annexin V-labeled cells among total thymocytes and DP cells on day 10 post-4-OHT injection. [*], *p* < 0.05.

Previously, we have taken a loss-of-function approach to conditionally ablate β-catenin and demonstrate that β-catenin expression in TECs is intrinsically required for TEC differentiation, thereby affecting thymocyte development and resulting in thymic atrophy [6]. In this study, we examined whether Tg1 mice exhibited leaky β-catenin activity, which might be able to compensate for the conditional ablation of β-catenin in K5-expressing TECs and cause thymic atrophy. Thus, we bred the Tg1 mice to the previously established *Ctnnb1^K5-fx/fx^* background to obtain *Tg1;Ctnnb1^K5-fx/fx^* mice. After 10 days of 4-OH-Tam (4-OHT) treatment, the size, cellularity, and CD4/CD8 distributions of *Tg1;Ctnnb1^K5-fx/fx^* adult thymi were similar to those of thymi from control animals (Figure 4A-4C). These data suggest that the expression of constitutively active β-catenin in Tg1 mice is sufficient to rescue *Ctnnb1^K5-fx/fx^* thymic atrophy. Furthermore, the *Ctnnb1^K5-fx/fx^* thymic phenotype, including the decreased frequency of proliferating thymocytes (total, CD4^−^CD8^−^DN and CD4^+^CD8^+^DP; Figure 4D) and the increased percentage of annexin V-labeled apoptotic cells (total and CD4^+^CD8^+^DP; Figure 4E), was significantly reversed by the transgenic expression of stabilized β-catenin in K5-expressing cells. Our data suggest that expression of the ΔN64Ctnnb1/ER^T2^ fusion protein in TECs can functionally complement the effects of β-catenin loss in the K5-expressing TEC lineage. This genetic complementation experiment also suggests that the ΔN64Ctnnb1/ER^T2^ fusion protein in TECs can compensate endogenous β-catenin function.

### Aberrant expression of AIRE and p63 in mouse thymomas is similar to that in human thymomas

**Figure 5:**
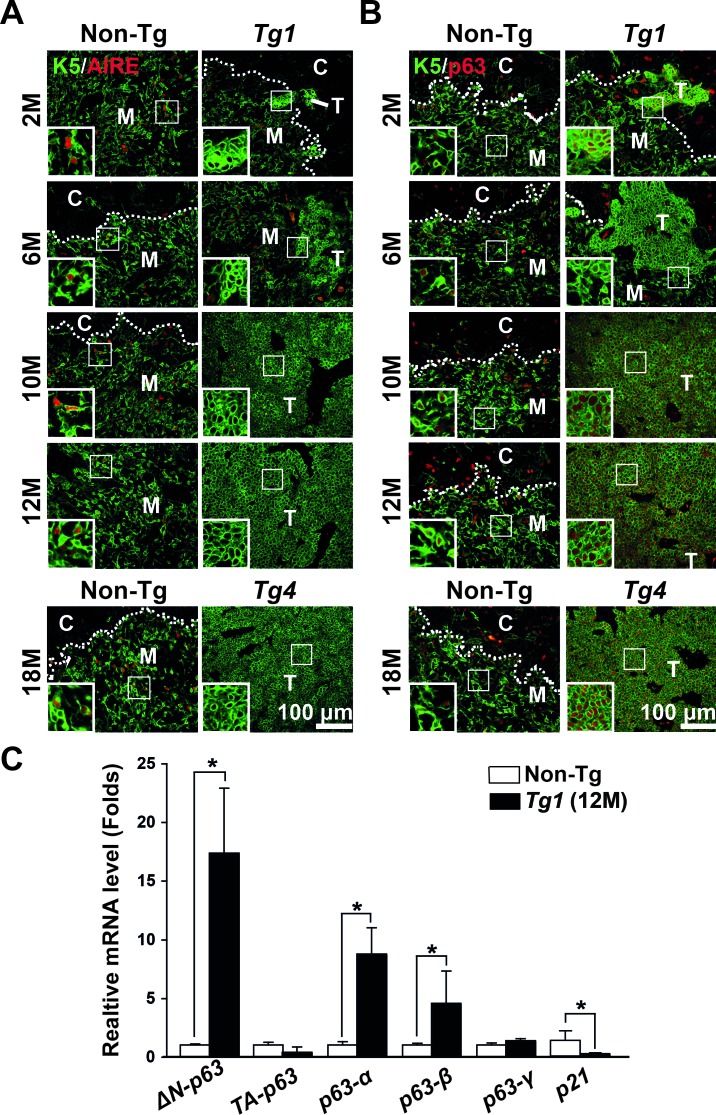
AIRE, p63 isoforms, and p21^waf1/cip^ expression in mouse thymomas **A.** Co-immunostaining of K5 (green) and AIRE (red) reveals scattered expression of AIRE in the K5-expressing cells of the non-Tg thymi, but loss of AIRE in the thymoma lesions of the Tg1 and Tg4 mice at the indicated time points. **B.** Co-immunostaining of K5 (green) and p63 (red) reveals scattered expression of p63 in the K5-expressing cells of the non-Tg thymi and uniformed expression of p63 in the thymoma lesions of the Tg1 and Tg4 mice at the indicated time points. Insects, high-power view of boxed areas; **C.** Bar graphs represent relative expression levels of p63 isoforms (TAp63, ΔNp63, p63-α, p63-β and p63-γ) and the p53/p63 target gene p21^waf1/cip^ in CD45^−^ thymic stromal cells of non-Tg thymi and in Tg1 thymomas (*n* = 3 in each group) at 12 months using RT-qPCR analysis. [*], *p* < 0.05.

The loss of AIRE [31] expression and intense p63 [32-34] expression have been reported in human thymomas and thymic carcinomas. Therefore, we determined the expression of AIRE and p63 in Tg1 thymoma samples and non-Tg thymi using co-immunofluorescence. We found that AIRE was undetectable in K5-expressing tumor cells of early thymoma lesions (2 or 6 months of age) or manifest thymomas of mice 10-12 months of age (Figure 5A). Comparatively, scattered AIRE expression was detected in medullary K5-expressing cells of the non-Tg thymi at the corresponding time points (Figure 5A). In contrast, p63 showed intense homogenous expression in the K5-expressing tumor cells of both early thymoma lesions and manifest thymomas compared to the scattered p63 expression in K5-expressing cells of the non-Tg thymi (Figure 5B). Similar expression patterns of AIRE and p63 were also observed in a Tg4 thymoma at 18 months (Figure 5A and 5B). Interestingly, loss of AIRE and presence of p63 were observed in Tg1 thymomas with leaky β-catenin activation without Tam treatment, but these expression patterns were also found in a short-term (3 days) treatment of Tam in Tg1 microscopic lesions (Supplementary Figure 5).

Next, we examined p63 transcripts in the manifest thymoma samples (*n* = 3) of Tg1 mice and purified CD45^−^ thymic stromal cells of non-Tg mice (*n* = 3) using RT-qPCR. Notably, p63 can be transcribed from two alternative promoters, producing TAp63 and ΔNp63 [35]. TAp63 and ΔNp63 may each have three C-terminal variants, called α, β and γ, that are generated by alternative splicing of 3′ exons [35]. Thus, we used isoform-specific primer sets to determine which p63 isoforms were expressed. Our results showed that ΔNp63, but not TAp63, was increased in the manifest thymoma samples compared to the non-Tg thymic stromal cells (Figure 5C). Furthermore, the spicing variants ΔNp63-α and -β, but not -γ, were upregulated in the manifest thymoma samples, suggesting that ΔNp63-α and -β were the major p63 isoforms involved with increased expression, which may contribute to tumorigenesis of TECs. Furthermore, one potential target gene of p53/p63 is p21^WAF1/cip1^, which can be suppressed by increasing levels of ΔNp63 isoforms [36-39]. We found that p21 transcript was significantly decreased in Tg1 thymomas compared to non-Tg CD45- thymic stromal cells (Figure 5C), a finding that is consistent with p21 downregulation in human thymomas [40].

### Aberrant expression of β5t and squamoid differentiation markers in the mouse thymomas

**Figure 6:**
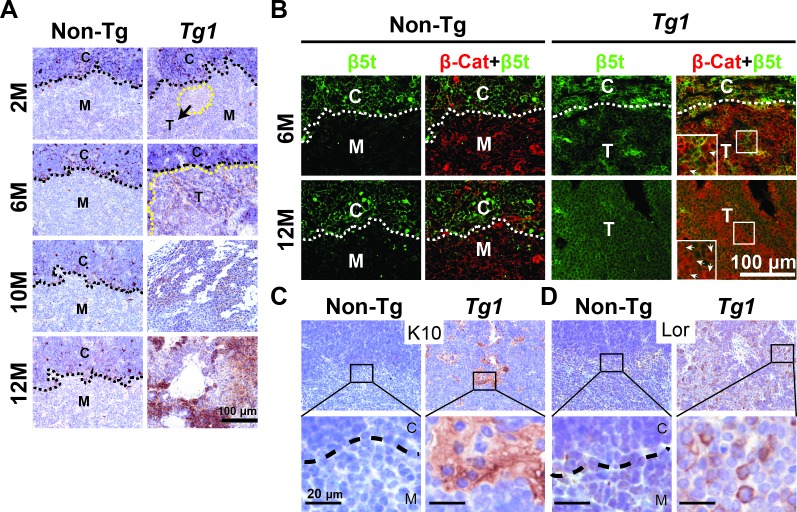
Aberrant β5t and squamoid differentiation markers expression in Tg1 mouse thymomas **A.** Immunohistochemistry reveals intense β5t expression (brown) in the cortex, but not in the medulla, of non-Tg thymi, and in the Tg1 thymoma lesions arising in the CMJ and medulla at 2, 6, 10, and 12 months. Hematoxylin is used for counterstaining. Black dashed lines represent the CMJ. Yellow dashed lines depict the thymoma boundary. **B.** Immunofluorescent staining of β5t (green) reveals β5t expression in cTECs of non-Tg thymi and in the Tg1 thymoma lesions at 6 and 12 months. Merged images of β5t (green) and β-catenin (red) co-immunostaining reveal that both proteins are co-expressed in the thymomas. Insets: high-power views of boxed areas that show stabilized β-catenin in both the cytoplasm and the nuclei (arrows); White dashed lines, CMJ; C, cortex; M, medulla; T, thymoma lesions. **C.** and **D.** Immunohistochemistry using anti-K10 and loricrin antibodies reveals focal K10 and scattered loricrin (lor) expression (brown) in the Tg1 thymomas compared to weak staining in the non-Tg thymi. Lower panels: high-power views of boxed areas in upper panels.

β5t is a cTEC-specific proteosomal subunit [41, 42] that has recently been used as a differential diagnosis marker of type B3 thymomas [43, 44]. To gain insight into the phenotypes of mouse thymomas developed in Tg1 mice, we examined the expression of β5t in Tg1 thymomas during initiation and progression. Using immunohistochemistry, we detected β5t in the cortical area in control thymi of Tg1 mice aged 2 to 12 months (Figure 6A). In 2-month-old Tg1 thymi, β5t was expressed in the cortical area similarly to non-Tg thymi and was weakly detected in focal microscopic lesions in medullary area (Figure 6A). As thymoma development proceeded, robust β5t expression was detected in most neoplastic cells from thymomas of 6- to 12-month-old Tg1 mice (Figure 6A). These findings suggest that β5t expression increased with age in Tg1 thymomas. This characteristic resembles that of type B3 thymomas in humans [43, 44]. Furthermore, we examined β5t and β-catenin expression in the early lesions in the thymi of Tg1 mice at 6 months of age and in manifest thymomas of Tg1 mice at 10-12 months of age. We found that the nuclear form of β-catenin and uniform expression of β5t were markedly increased in manifest at 12-month-old thymomas compared with that of the early lesions at 6 months of age (Figure 6B). Thus, transgenic mice expressing stabilized β-catenin in TECs exhibit thymoma development and molecular characteristics, such as β5t, that resemble that of type B3 thymomas in humans.

While previous studies showed that Apc loss or β-catenin stabilization in TECs caused squamous metaplasia in the developing thymus [26, 28], no thymoma phenotype was reported. To characterize TECs-derived thymomas in Tg1 mice, the expression of squamoid differentiation markers K10 and loricrin [45, 46] were used in immunohistochemistry. We found focal positive for K10 expression (Figure 6C) and scattered positive for loricrin expression (Figure 6D) in manifest thymomas from 12-month-old Tg1 mice. The heterogenously expressed K10 and loricrin indicated aberrant squamoid cell-fate differentiation in some neoplastic cells of Tg1 thymomas.

## DISCUSSION

In this study, the transgenic mice expressing *ΔN64Ctnnb1/ER^T2^* under the control of K5 promoter enable us to monitor thymoma progression from early lesions to manifest thymomas. These thymomas have histological and molecular characteristics that resemble those of type B3 thymomas in humans. Our findings also suggest that induced β-catenin activation is sufficient to drive thymoma initiation, forming microscopic lesions. Loss of AIRE expression and gain of ΔNp63-α and -β isoform expression in the microscopic lesions, as well as in the manifest thymomas, appear to be common features between this mouse model and human thymomas [31-34]. Interestingly, thymomas developed in the Tg1 mice also express β5t, which is a differential diagnostic marker for type B3 thymomas in humans [43, 44]. Both K10 and loricrin were focally expressed in some neoplastic cells in Tg1 thymomas, suggesting that over-expression of the ΔN64Ctnnb1/ER^T2^ fusion protein in TECs may adopt squamous cell-fate differentiation.

Although the molecular genetics of human thymomas are largely unclear, the presence of *APC* mutations has been linked to pathogenesis of type B3 thymomas [47]. Also, LOH at *APC* locus has been found in the type B thymomas [24, 25]. These reports implicate the possible link of APC-controlled β-catenin activation in human thymomas. Previously, *Apc* conditional ablation by K14-Cre mice showed aberrant growth of epidermal-derived organs and squamous metaplasia in the thymus [26]. However, these *Apc*-conditional knock-out mice (*K14-Cre;Apc^CKO/CKO^*) die before weaning, thus precluding follow-up studies to monitor thymic homeostasis and thymoma development. In our study, the transgenic mice expressing the ΔN64Ctnnb1/ER^T2^ fusion protein exhibited leaky β-catenin activation in K5-expressing cells, but this phenotype did not lead to early lethality, thus enabling us to monitor thymoma development as the transgenic animals aged. Induction with tamoxifen initiated thymoma lesions in a temporally control manner, enabling us to examine molecular changes in the early stages of thymoma progression. The thymomas that developed in the Tg1 and Tg4 mice reflect the expression levels of the ΔN64Ctnnb1/ER^T2^ transgene and share histological appearance (e.g. cellular atypia and perivascular space) and expression patterns of cellular markers (AIRE, p63, p21 and β5t) with those of human thymomas. Therefore, these findings suggest that the β-catenin activation is possible to drive thymoma pathogenesis.

Genetically engineered mouse models are extremely valuable for the modeling of human diseases because they enable us to validate causative mediators using both loss- and gain-of-function approaches. Diseases such as thymoma, which have various clinically unsolved problems, are difficult to tackle because of the rarity of clinical cases reported. Previously, SV40 T-antigen, driven by its own viral early region elements, which include an enhancer and a promoter, caused thymic epithelial carcinomas in transgenic mice [48]. Additionally, forced expression of the E6/E7 oncogene driven by the K5 promoter caused thymic epithelial tumors in the transgenic mice [49]. These viral oncogenes are commonly used for transgenic studies to block p53 or Rb checkpoints in the cell cycle, leading to tumorigenesis [50]. In addition, overexpression of E2F2, which is a E2F member and downstream target of Rb, alters cell cycle progression [51] in transgenic mice, causing a high incidence of thymomas [52]. These transgenic mouse models suggest that dysregulated cell cycle control can be the critical determinant for thymoma development. Consistently, the levels of cell cycle regulators p21, p27, and p53 have been examined in encapsulated thymomas in a clinical study [40]. Increased p53 and decreased p21 and p27 in thymoma tissue correlated with poor prognosis for shorter disease-free survival [40], suggesting that aberrant regulation of cell-cycle progression contributes to the clinical outcomes of the patients with thymomas. In this study, we also demonstrated downregulated p21, which was coincidently associated with upregulated ΔNp63-α and -β isoforms, which may work together to promote TECs tumorigenesis.

In summary, our study provides data to generate a fundamental basis for understanding the activation of β-catenin in the progression of thymic epithelial carcinogenesis. In the future, our mouse models may be used as the *in vivo* platforms for validating the therapeutic agents and strategies to treat the thymomas.

## MATERIALS AND METHODS

### Mice and genotyping

*Tg(K5-ΔN64Ctnnb1/ER^T2^)* transgenic mouse lines Tg1, Tg4, and Tg10 were generated on the C57BL/6 background for this study. The *K5-ΔN64Ctnnb1/ER^T2^* transgenic plasmid (Figure 1A) was composed of the bovine keratin 5 promoter (derived from BK5-Cre plasmid, which was kindly provided by Dr. José L. Jorcano), a rabbit β-globulin intron sequence (IVS), a Myc-tagged sequence, two copies of a nuclear location signal (NLS), a portion of the *Ctnnb1* encoding N-terminal 64-amino acid deletion mutant of β-catenin, the mutated ligand-binding domain of human estrogen receptor (*ER^T2^)* gene (derived from Cre-ER^T2^ plasmid, which was kindly provided by Dr. Pierre Chambon, IGBMC, France), an SV40 polyadenylation signal (pA), and two copies of the *HS4* insulators. The 11.4-kb *Not*I/*Sal*I-digested transgene fragment was eluted and separated from the *pBluescript* vector backbone for pronuclear microinjection. TOP-Gal transgenic mice [53] were obtained from the RIKEN biological resource center (Experimental Animal Division, RIKEN BRC, Ibaraki, Japan), and then bred with Tg1 to generate Tg1;TOP-Gal mice. *Ctnnb1^K5-fx/fx^* mice carrying the *BK5-CreER^T^* transgene [54] and the *Ctnnb1* conditional allele [55] were described previously [6]. Tg1;*Ctnnb1^K5-fx/fx^* mice were generated by crossing *Ctnnb1^K5-fx/+^* with *Tg1;Ctnnb1^fx/+^* mice. Allelic specific genotyping was conducted by PCR methodology. The *Tg(K5-ΔN64Ctnnb1/ER^T2^)* transgene was detected using the Myc-tag forward primer (5′-gct cat ttc tga aga gga ctt g-3′) and Ctnnb1 reverse primer (5′-gct cag gaa ttg cac gtg tgg-3′), yielding a 400-bp PCR product. The TOP-Gal transgene was detected with forward primer (5′-ttg ccg tct gaa ttt gac ctg-3′) and reverse primer (5′-tct gct tca atc agc gtg cc-3′), yielding a 500-bp PCR product. Genotyping of *Ctnnb1^K5-fx/fx^* mice was described previously [6]. All experiments with mice were performed with the approval of the Institutional Animal Care and Use Committee (IACUC) at National Yang-Ming University.

### Tamoxifen administration

Tamoxifen (Tam) (Sigma-Aldrich, St. Louis, MO, USA) was dissolved in 100% ethanol (10 mg/ml) and then mixed in sunflower seed oil (oil; Sigma-Aldrich) before intraperitoneal injection. Mice at 8 weeks of age were injected with Tam (0.5 mg in 100 μl oil) or vehicle (oil) only for 5 consecutive days. 4-OH-Tam (4-OHT) was dissolved in DMSO and then emulsified in oil as described previously [6, 54, 56]. Mice at the 8 weeks of age were injected intraperitoneally with three doses of 4-OHT (0.1 mg in 250 μl oil) every other day.

### Western blot assay

Generally, the experimental procedures of western blot assay were completed as described previously [56]. Primary antibodies against β-catenin (mouse IgG. 1:1000; BD Bioscience) and GAPDH (mouse IgG, 1:2000; Santa Cruz) were used for these analyses.

### Flow cytometry and sorting of TECs

Experimental procedure was performed as described previously [6]. Briefly, the thymic fragments dissected from non-Tg and Tg mice were treated with collagenase (1 mg/mL collagenase D; Sigma) and DNase I (0.1 mg/mL; Sigma) at 37°C for 15 minutes. Subsequently, collagenase D activity was terminated by adding phosphate buffered saline (PBS) containing 1% FBS and 0.5 mM EDTA. In addition, the portion of Tg1 thymomas that could not be completely dissociated by collagenase was collected and subsequently treated with Trypsin (0.05%)-EDTA(0.02%) (Invitrogen) for preparing the single cell suspension. Finally, the cell suspension were washed with PBS and then stained with APC-congujated anti-CD45 antibody (1:200; Biolegend, San Diego, CA, USA), PE/Cy7-conjugated anti-EpCAM antibody (1:400; eBiocience, San Diego, CA, USA), Rhodamine-conjugated UEA1 (1:50; Vector Laboratories, Burlingame, CA, USA) and FITC-conjugated anti-Ly51 antibody (1:400; Biolegend) for the following flow cytometry analysis by BD FACScalibur (BD Biosciences). For cell sorting experiment, the UEA1^+^ and Ly51^+^ subsets were gated and sorted from CD45^−^EpCAM^+^ TEC populations by a BD FACSAria cell sorter (BD Biosciences).

### Quantitative RT-PCR (RT-qPCR)

Detailed experimental procedures were described previously [6]. Expression of *ΔN64Ctnnb1/ER^T2^* was detected by the transgene-specific Myc-NLS primers (5′-GAG CAA AAG CTC ATT TCT GAA-3′and 5′-TAC TTG CTC TTG CGT GAA GG-3′) or ER^T2^ primers (5′-CTT GCT CTT GGA CAG GAA CC-3′ and 5′-CGA GAT GAT GTA GCC AGC AG-3′). Expression of K5 was detected using primers 5′- ACA GGA AGC TGC TGG AGG GC-3′ and 5′-GGT GGA GAC AAA TTT GAC ACT GG-3′. Expression of Aire was detected using primers 5′-GGT TCC TCC CCT TCC ATC-3′ and 5′-GGC ACA CTC ATC CTC GTT CT-3′. Expression of β5t was detected by primers 5′-GCT GTA TAG GGA GCT GCA GAA-3′ and 5′-TGG CAG GCT CAG GAT AGA TT-3′. Expression of Axin2 was detected using primers 5′-ctg ctg gtc agg cag gag-3′ and 5′-tgc cag ttt ctt tgg ctc tt-3′. Expression of Ccnd1 was determined using the primers 5′-ctg gcc atg aac tac ctg ga-3′ and 5′-gtc aca ctt gat cac tct gg-3′. Expression of c-Myc was determined using the primers 5′-cga aac tct ggt gca taa act g-3′ and 5′-gaa ccg ttc tcc tta gct ctc a-3′. Expression Δ*N*p63 and TAp63 isoforms was detected using a common reverse primer 5′-gag gag ccg ttc tga atc tg-3′ with the forward primers 5′-caa aac cct gga agc aga aa-3′ or 5′-gtg gat gaa cct tcc gaa aa-3′, respectively. The alternatively spliced p63-α isoform was detected using primers 5′-cac tct cca tgc cct cca-3′ and 5′-gcc caa cct tgc taa gaa act-3′. The other alternative splice variants, p63-β and -γ, were detected using common forward primer 5′-tcc ctc agc aca cga tcg a-3′ and specific reverse primers 5′-act tgc caa atc ctg aca atg c-3′ and 5′-gac gtc aga ctg tgt cgg agc-3′, respectively. Expression of *p21^WAF1/CIP1^* was detected using the primers 5′-ttg cca gca gaa taa aag gtg-3′ and 5′-ttt gct cct gtg cgg aac-3′. Ribosomal RNA L19 was used as internal control and detected with the primers 5′-tcg ttg ccg gaa aaa cac-3′ and 5′-agg tca cct tct cag gca tc-3′.

### Characterization of thymoma phenotypes

Hematoxylin & eosin (H&E) staining, immunofluorescence and immunohistochemistry were conducted to characterize the thymomas developed in the Tg1 and Tg4 mice. The detail experimental procedures for the tumor phenotype characterization have been described in our previous studies [6, 56-58]. The primary antibodies against K5, K8, β-catenin, AIRE, and p63 were described previously [6]. Anti-β5t antibody (rabbit IgG, Medical and Biology Laboratories, Nagoya, Japan) was diluted at 1:400 for both immunohistochemistry and immunofluorescence staining in this study. Anti-K10 (rabbit IgG, Abcam, Cambridge, UK) and anti-loricrin (rabbit IgG, Zymed Laboratories, South San Francisco, USA) were diluted at 1:200 and 1:1000, respectively, for immunohistochemistry.

### Expression and activation of TOP-Gal

The experimental procedures of whole-mount X-gal staining were described previously [54, 58, 59]. The antibody against β-galactosidase (rabbit IgG, 1:1000; MP Biomedicals, Santa Ana, California, USA) used in immunostaining was performed as described previously [60].

### Flow cytometry analysis of thymocytes

CD4/CD8 profiles, BrdU labeling, and Annexin V-binding of thymocytes were conducted by flow cytometry analysis as described in a previous study [6].

### Statistical analysis

All statistical data were analyzed using the paired Student's t-test for statistical comparisons. *P* < 0.05 was defined as statistically significant.

## SUPPLEMENTARY MATERIAL FIGURES


