# Supplementary material for: β-catenin activation drives thymoma initiation and progression in mice
Source: Oncotarget. 2015 Jun 8;6(16):13978–93. doi: 10.18632/oncotarget.4368 (PMC4546445; doi:10.18632/oncotarget.4368)
Supplement: Supplementary file 1 [file oncotarget-06-13978-s001.pdf]

β-catenin activation drives thymoma initiation and progression in mice

Supplementary Material

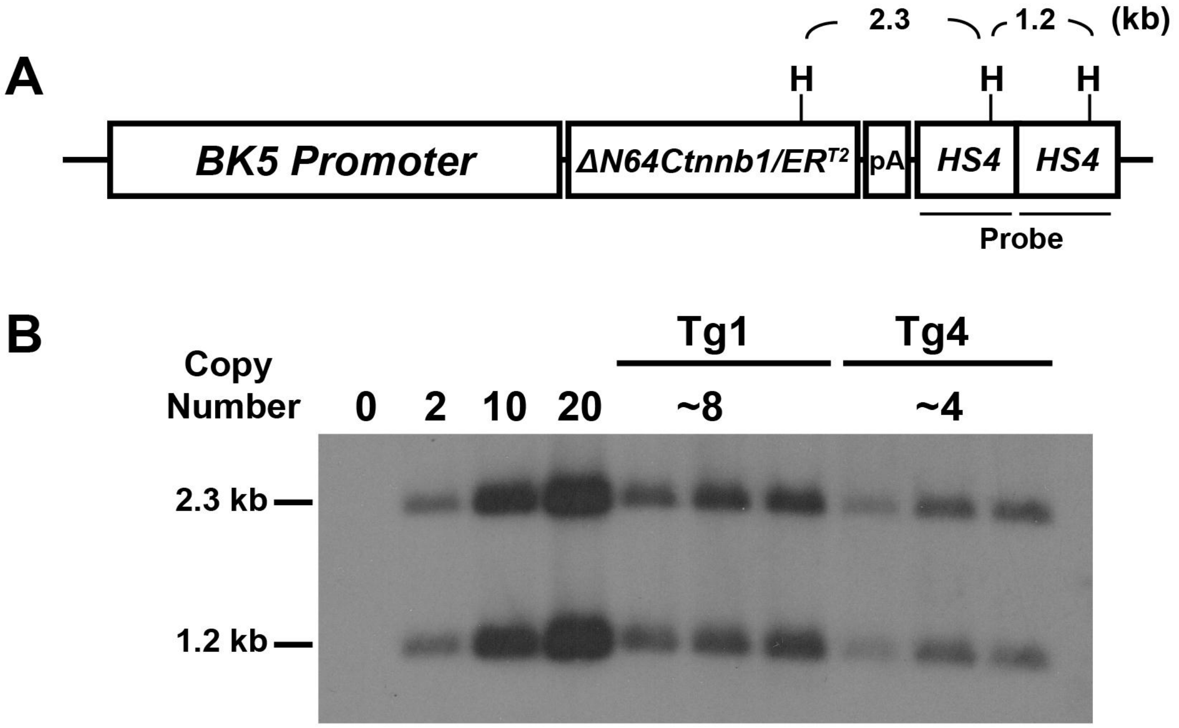

Supplementary Figure 1: Characterization of transgenic copy number of Tg1 and Tg4 mice. (A) Diagram illustrates the Hind III sites on the transgenic construct. The DNA fragment harboring insulator (HS4) was used as the probe. (B) Southern blot analysis reveals a 2.3-kb and a 1.2-kb Hind III-digested DNA fragments hybridized with the HS4 probe. The copy number of Tg1 and Tg4 mice (n = 3, each line) was quantified by the intensity of their Hind III-digested genomic DNA (3 µg) compared with the same amount of B6 wild-type genomic DNA mixed with 0, 2 (11.4 pg), 10 (57 pg), and 20 (114 pg) copies of transgenic DNA. Approximately, 5.7 pg of *K5-ΔN64-Ctnnb1/ER*^{T2} transgenic DNA is calculated as one copy of transgene that is equivalent to 3 µg of a diploid mouse genome.

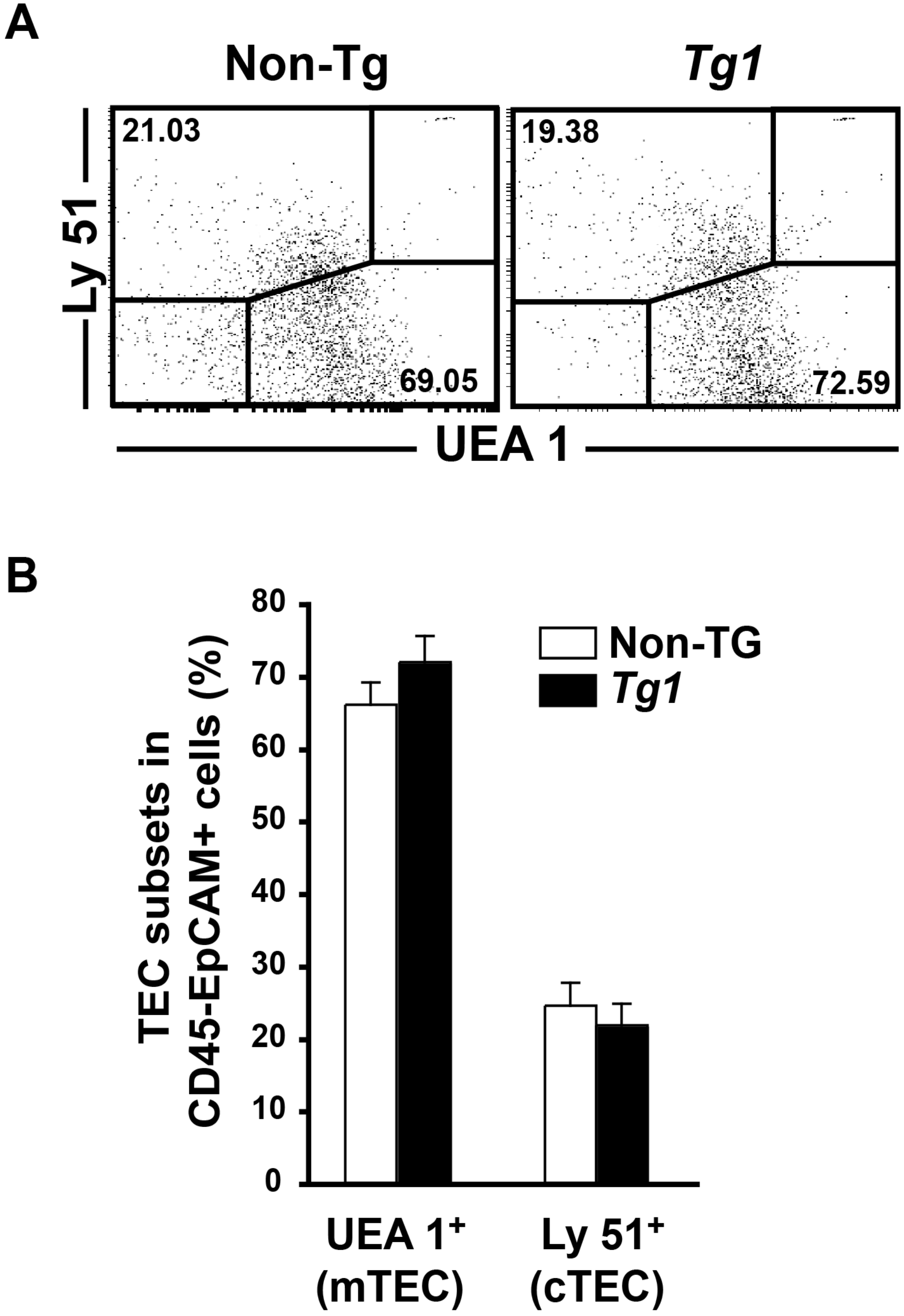
A
Non-Tg
Tg1
21.03
69.05
19.38
72.59
Ly 51
UEA 1
B
TEC subsets in CD45-EpCAM+ cells (%)
0
10
20
30
40
50
60
70
80
Non-TG
Tg1
UEA 1+ (mTEC)
Ly 51+ (cTEC)

Supplementary Figure 2: Flow cytometry analysis of mTECs and cTECs in the thymi of Tam-treated non-Tg and Tg1 mice. (A) Representative dot-plots of flow cytometry analysis based on the surface markers, UEA1 and Ly51, in $CD45^{-}EpCAM^{+}$ gated TECs of the thymi of Tam-treated 8-week-old non-Tg and Tg1 mice. (B) Bar graphs represent the percentages of $UEA1^{+}Ly51^{-}$ mTECs and $UEA1^{-}Ly51^{+}$ cTECs in the thymi of non-Tg and Tg1 mice (n = 3, each group) after Tam administration for 3 days. These cells were sorted for RT-qPCR experiments as shown in **Figure 1C**.

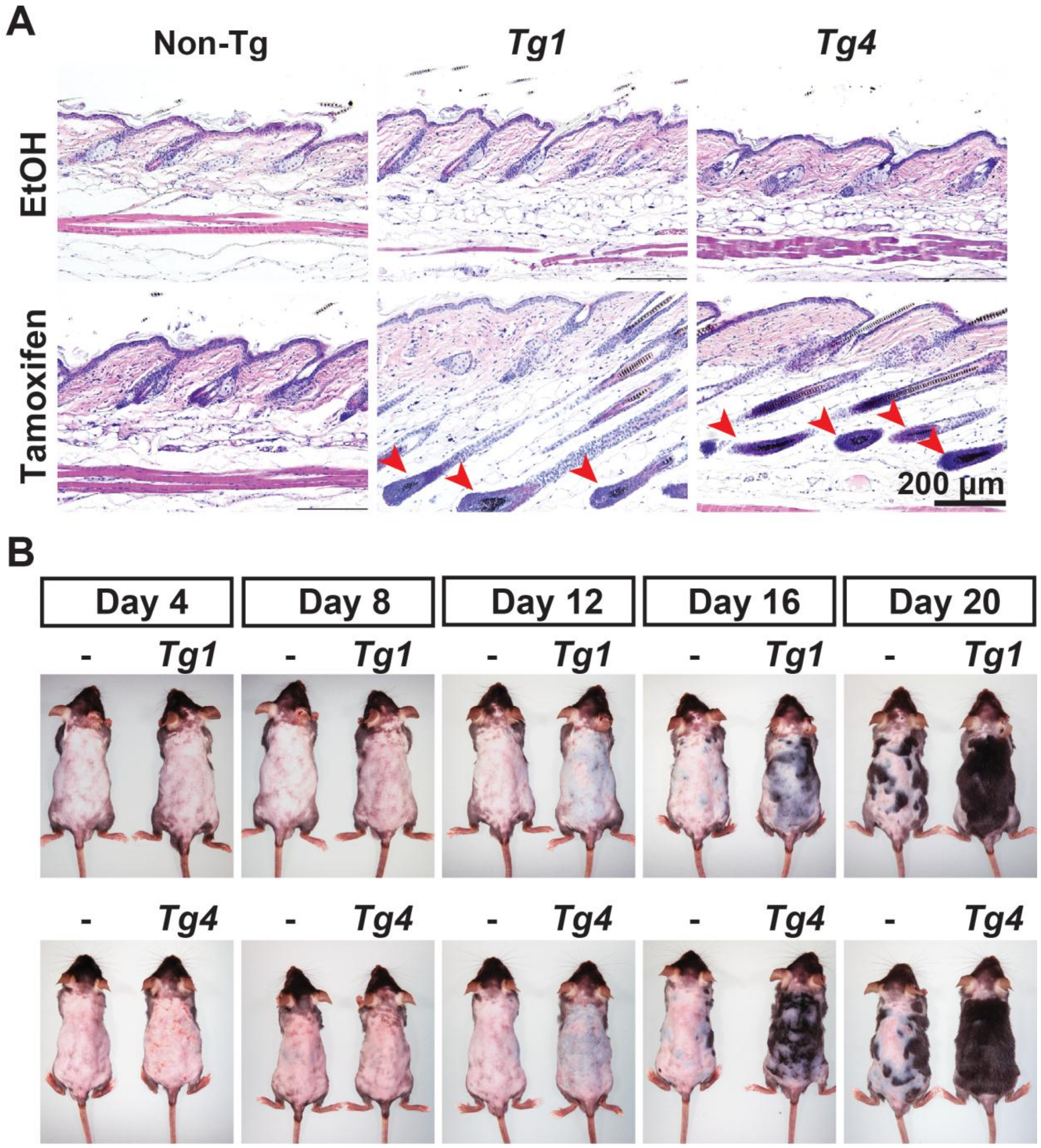

Supplementary Figure 3: Inducible and spontaneous ΔN64Ctnnb1/ER^{T2} activation in the hair cycle re-entry. (A) H&E staining shows telogen hair follicles in ethanol-treated groups (non-Tg, Tg1, and Tg4 skins) and Tam-treated non-Tg skin. In contrast, hair cycle re-entry to anagen follicles (arrowheads) can be observed in Tam-treated Tg1 and Tg4 skins. (B) Representative data for comparison of hair re-growth in the non-Tg (-), Tg1 and Tg4 mice (n = 3 each group) after hair shaved for 4, 8, 12, 16, and 20 days.

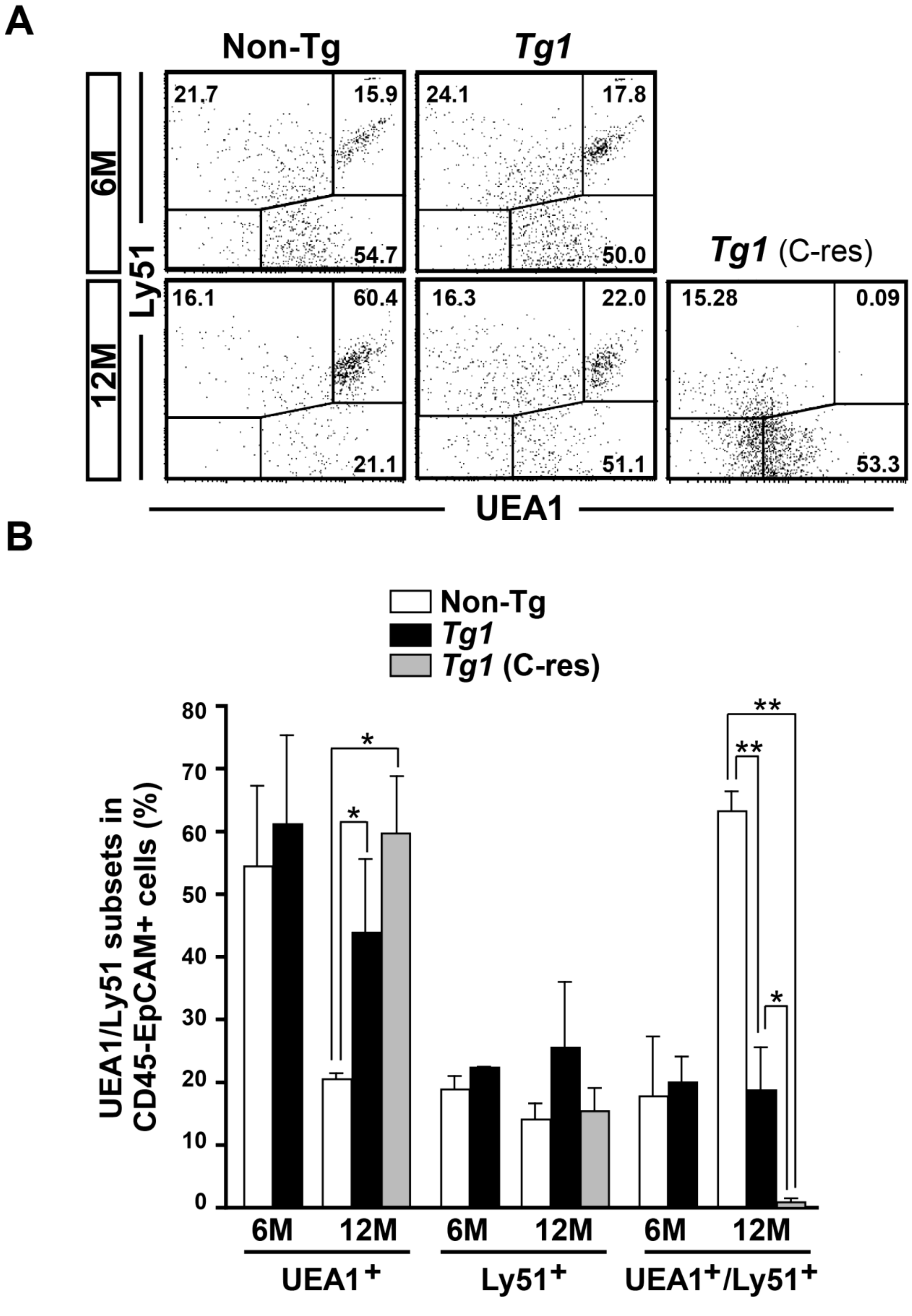
A
Non-Tg
Tg1
Tg1 (C-res)
6M
12M
Ly51
UEA1
21.7
15.9
54.7
24.1
17.8
50.0
16.1
60.4
21.1
16.3
22.0
51.1
15.28
0.09
53.3
B
Non-Tg
Tg1
Tg1 (C-res)
UEA1/Ly51 subsets in CD45-EpCAM+ cells (%)
80
70
60
50
40
30
20
10
0
6M
12M
UEA1+
Ly51+
UEA1+/Ly51+
*
**

Supplementary Figure 4: Phenotypic characterization of Tg1 thymomas using flow cytometry analysis. (A) Representative dot-plots of flow cytometry analysis based on the surface markers, UEA1 and Ly51, in $CD45^{-}EpCAM^{+}$ gated TECs of the non-Tg thymi and Tg1 thymomas at 6 and 12 months of age. Percentages of three major subsets, $UEA1^{+}Ly51^{-}$, $UEA1^{-}Ly51^{+}$, and $UEA1^{+}Ly51^{+}$, are indicated in the gated areas. A portion of thymomas exhibited collagenase resistance, which was referred to as C-res. (B) The percentages of $UEA1^{+}Ly51^{-}$, $UEA1^{-}Ly51^{+}$, and $UEA1^{+}Ly51^{+}$ subsets in the thymi of non-Tg and the thymomas of Tg1 mice at 6 (n = 6) and 12 months of age (n = 3). [*], $p < 0.05$; [**], $p < 0.001$.

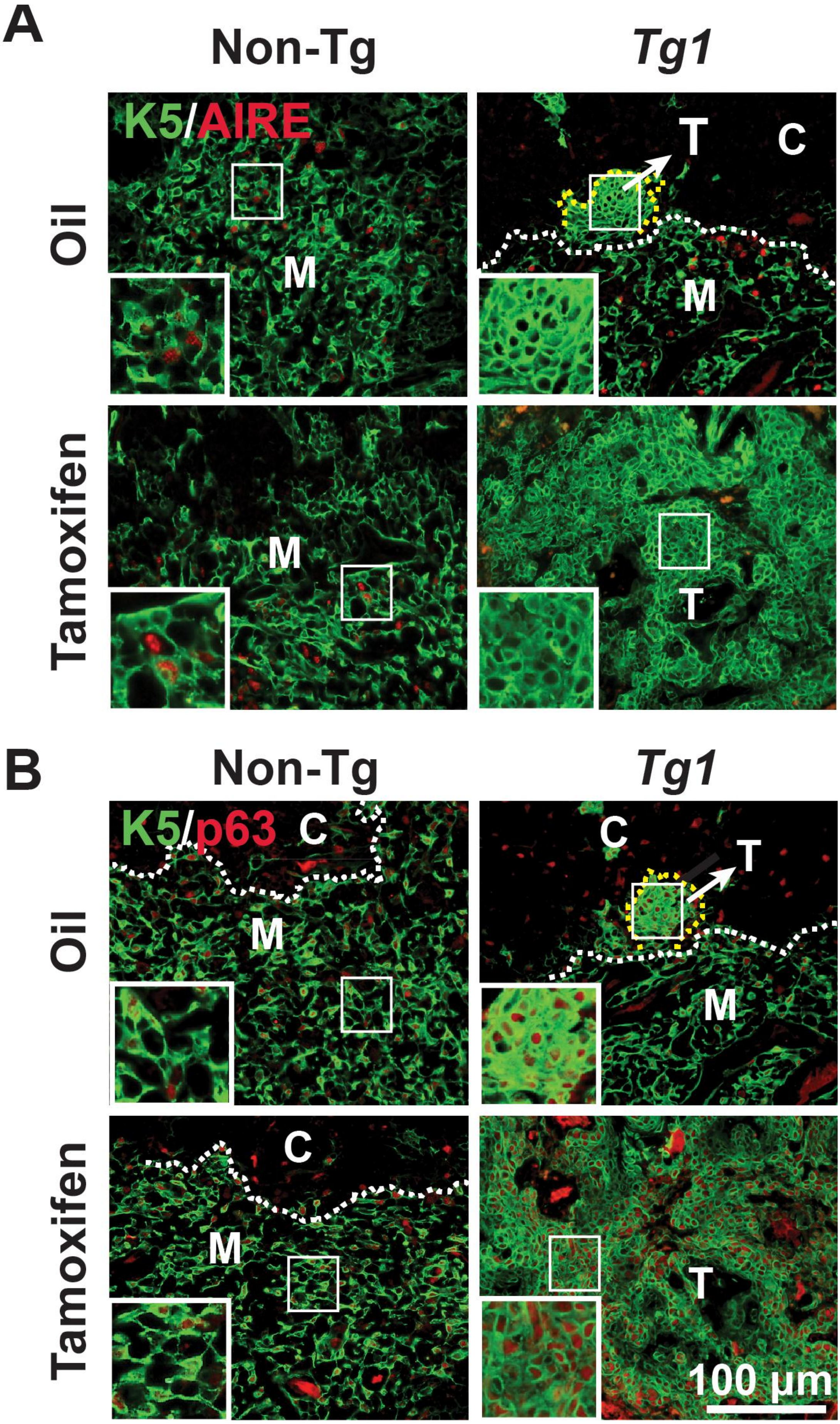
A
Non-Tg
Tg1
K5/AIRE
Oil
Tamoxifen
M
T
C
B
Non-Tg
Tg1
K5/p63
C
M
T
100 µm

Supplementary Figure 5: Microscopic thymoma lesions with loss of AIRE and expression of p63 in the short-term oil- or Tamoxifen-treated Tg1 thymi. (A) Co-immunofluorescent staining of K5 (green) and AIRE (red) reveals a scattered expression pattern of AIRE in the K5-expressing TECs of the non-Tg controls and loss of AIRE expression in the microscopic thymoma lesions of the Tg1 thymi, in the absence and presence of Tam for 3 days. (B) Co-immunofluorescent staining K5 (green) and p63 (red) reveals a scattered expression pattern of p63 in the K5-expressing TECs of the non-Tg control and uniform p63 expression in the microscopic thymoma lesions of the Tg1 thymi in the absence or presence of Tam for 3 days.
